# Toward Technology-Based Education and English as a Foreign Language Motivation: A Review of Literature

**DOI:** 10.3389/fpsyg.2022.870540

**Published:** 2022-04-29

**Authors:** Yi Wei

**Affiliations:** Foreign Languages Department, Shijiazhuang Tiedao University, Shijiazhuang, China

**Keywords:** computer assisted language learning (CALL), educational technology, mobile assisted language learning (MALL), motivation, learners’ grit

## Abstract

This review examined the studies on the role of technology-based English as a foreign language (EFL) academic motivation. A significant positive correlation between academic motivation and educational technology use has been approved in related studies. However, there is a dire need for studying the effect of Mobile-Assisted Language Learning (MALL) and Computer-Assisted Language Learning (CALL) on learners’ motivation. The literature showed that purposeful attractiveness, effectiveness, and usefulness of digital instruments can positively affect learner motivation. There are also some reasons for increasing learner motivation in educational technology contexts, such as learners’ integration with the community, familiarising with different societies and cultures, input flooding opportunities, engagement in academic contexts, and interaction with native speakers. In the end, the paedagogical implications are expounded to promote the learners’ grit and diminish anxiety for better performance. This review also provides suggestions for further research to clarify our perspective on emotional variables like motivation.

## Introduction

Recently, a large number of online technology developments and the ever-growing expansion of electronic devices have provided language learners with pervasive and genuine language input. Modern technologies have supported English as a foreign languages (EFLs) to participate in the educational context of language learning (Rassaei, 2017). Lately, numerous investigations have examined the impacts of integrating various technologies in education (Benson and Chik, 2011). Face to screen language learning has a key role in developing language competencies (Fathali and Okada, 2018). Integrating technology into classroom instruction involves more than just teaching computer skills. It demands that educators look for means of innovation to encourage students’ motivation and build up their learning. Therefore, one way to accomplish this important aim is the use of instructional technology in an effective way.

Foreign language learners’ emotional states, like motivation, have played influential roles in foreign language learning. Motivation is normally characterised as a learner’s “willingness or desire to be engaged in or commit effort to complete a task” [Zhou (2012), p. 1318]. Without the desire to learn, students are less likely to co-operate, take self-responsibility, or fully engage in the language learning process. Dörnyei (2001) stated that motivation is regarded as a critical emotional state that affects foreign language learning achievement. Many investigations have been done on the influence of various kinds of motivation on academic performance (e.g., Arabmofrad et al., 2019; Karabatak and Polat, 2020; Mammadov et al., 2021). If we want our learners to put up with the prolonged procedure of foreign language learning and foster the academic motivation required for learning and understanding English as a foreign language, then we are required to create proper methods of instruction (Dörnyei, 2001). This review investigates the related literature toward using technology in the classrooms and its effect on learners’ motivation. In addition, a significance of the current review is the adaptation of digital tools in different contexts of language learning.

## Literature Review

### Technology in Education and Its Effectiveness

The digital age and its modern technologies have considerably transformed the way of communication among individuals (Yadav et al., 2018). Nowadays, technology, with its continuous developments, has changed learning strategies and teaching methodologies (Hollands and Escueta, 2019). It offers numerous opportunities for EFL learners to simply communicate with native speakers in a foreign language context (Reinders and Benson, 2017). The integration of technology in the educational context has brought promising opportunities for instructors and learners to increase the efficiency of the paedagogical process (Yenkimaleki and van Heuven, 2019). In this respect, Spector and Yuen (2016) defined educational technology as “a theory and practice of designing, producing, using, and evaluating learning processes and resources” (p. 17). Zengin and Aksu (2017) stated that education has been affected by technological developments, such as computers, the internet, emails, mobile applications, and digital games. Butler-Pascoe (1997) also stated that “using technology, such as computer, plays a big role as a multi-sensory collection of text, sound, pictures, video, animation, and hypermedia to provide meaningful contexts to facilitate comprehension” (Butler-Pascoe, 1997, p. 20). Hence, it can be said that it is generally approved that digital tools are influential in learning achievement.

Computers and the internet help learners to communicate efficiently in educational contexts. Levy (1997) defined computer-assisted language learning (CALL) as “the study of applications of the computer in language teaching and learning” (p. 1). Some studies have been done on the role of technology in education in learners’ language skills and their academic achievements. Bensalem (2020), in a study on the effect of CALL on learners’ language skills, found out that the integration of technological developments, such as the internet, can improve learners’ reading skills and contribute to efficient communication of EFL learners. He argued that educational technology, in contrast with traditional face-to-face education, can reduce numerous challenges like shyness and apprehension. In addition, it can decrease learner differences in terms of learning style strategies which are fundamental impediments in traditional learning contexts. Likewise, Hassan Taj et al. (2017) found out that computers, particularly email and the internet, can improve learners’ L2 reading skills and vocabulary knowledge. They argued that digital tools can develop EFL learners’ working memory to retain and recall word meanings.

Moreover, mobile-assisted language learning (MALL) has provided new prospects for EFL educators to increase collaboration and performance in learning language skills (Godwin-Jones, 2011). Mobile social applications, including Facebook, Twitter, YouTube, Instagram, and Flicker, and Course Management Service (CMS), Mobile Podcasting, and Automatic Speech Recognition (ASR) are very significant in language performance and skills (Kim and Kwon, 2012). Alshammari et al. (2018) investigated the effect of MALL on EFL learners’ reading comprehension. Using WhatsApp as a tool in EFL instruction, they found out that online EFL learners outperform in reading comprehension. They also argued that technology changes teacher-learner rapport and their roles in an educational context. Wu (2015), in a study, revealed that mobile devices are critical in providing opportunities for language learners’ achievement in vocabulary and writing skills. However, Sadeghi and Dousti (2013) stated that it is not easy to investigate the effectiveness of CALL and MALL on language learners’ performance. They argued that the efficiency of CALL and MALL programmes depends on the proper use of the programme. Moreover, the efficiencies of CALL and MALL programmes are influenced by intervening moderator variables, including individual differences, task complexity, and instructional environment.

Educational technology affects and challenges learners’ positive and negative emotions (Guri-Rosenblit, 2018). For example, learners’ grit can be affected by online educational contexts. For instance, McClendon et al. (2017) stated that some non-cognitive factors, such as grit, persistence, and mindsets, are significantly affected by online learning in technological education. They argued that grit, mindset, and persistence increase learners’ engagement in online classes and improve learner achievement. Lan and Moscardino (2019) argued that grit was regarded as an essential component of educational success in a COVID-19 pandemic and was considered a psychological procedure that triggered and guided individuals’ activities in online learning contexts. Nussbaum et al. (2020) emphasised the role of critical thinking, creativity, heuristic evaluation, and grit in the development of online language learning contexts. Liu et al. (2021) studied grit in mobile learning contexts among EFL learners. They found that grit has a significant relationship with academic performance in mobile EFL learning. Furthermore, educational technology can foster learner engagement (Chiu, 2021). Golonka et al. (2014) asserted that the primary objective of technology is to engage learners and improve social interactions with peers. Khojah and Thomas (2021) found that task designs *via* digital technologies in educational contexts can raise learner engagement in language skills. Günüç and Kuzu (2014) also stated that teachers and learners can engage in classroom context by using technology. Regarding negative emotions, Dogan (2020) investigated the effect of online and traditional learning on learners’ foreign language anxiety (Ushida, 2005). In a study, Warschauer (1996) found that learners are inclined to be anxious in an online context. He found two levels of anxiety among learners: foreign language anxiety and online learning anxiety. He argued that a difference between a learner’s anticipation, experience, and apprehension from technology among learners can justify the reason for learners’ foreign language anxiety and online learning anxiety. However, the use of technology in EFL educational contexts helps learners not only to learn a foreign language, but also to increase their motivation (Abdulrahman and Basalama, 2019). This review tries to ponder into the relationship between technology use in an educational context and learners’ motivation.

### The Concept of Academic Motivation

Motivation is a widely studied issue in foreign language learning investigations. Gardner (1985) defined motivation as a “combination of effort plus desire to achieve the goal of learning the language” (p. 254). Furthermore, Gredler et al. (2004) pointed out that motivation is “the attribute that moves us to do or not to do something” (p. 106). Csizér and Dörnyei (2005) also stated that “motivation is a concept that explains why people behave as they do rather than how successful their behaviour will be” (p. 20). It guides various learning behaviours in numerous educational environments (Boo et al., 2015). Dornyei and Ushioda (2011) regard motivation as a component of enjoyment. In addition, they mentioned that motivation urges individuals to decide, be involved in action, and to attempt and carry on tasks. A learner is regarded as motivated when he/she tends to persistently reach objectives and puts his/her best effort to get them by using approaches and strategies (Lovato, 2011).

However, Dörnyei (2001) stated that there is an indirect relationship between academic motivation and language learning achievements due to other variables, such as linguistic skills and linguistic backgrounds, that affect learners’ proficiency in foreign language contexts. However, he also mentioned that some problems that occurred in school and classroom environments demotivated them. Alrabai and Moskovsky (2016) indicated that L2 learning proficiency is affected by motivation, self-sufficiency, perspective, nervousness, and self-confidence. They stated that academic motivation, compared to other variables, is significantly correlated with language learning proficiency. Crookes and Schmidt (1991) underscored the significance of motivational elements stated by Keller (1983), namely, (a) interest, (b) relevance, (c) expectancy, and (d) satisfaction. They specified that motivational elements should be highlighted by teachers when they attempt to understand learners’ motivation as these elements may influence learners’ language learning (Crookes and Schmidt, 1991).

Two foremost components of L2 learning motivation are called integrative and instrumental motivations (Fan and Feng, 2012; Carrió-Pastor and Mestre, 2014; Quan, 2014). Gardner (2010) stated that motivation is a combination of internal and external elements that inspire students to engage in the learning process. He also asserted that learners’ integrative motivation refers to their inclination to incorporate themselves into a target language society. Moreover, learners’ positive perspectives toward the educational context specify their integrative motivation and academic achievement (Khodadad and Kaur, 2016). Pavelescu (2019) found out that learners’ integrative motivation is affected by teachers’ motivation, encouragement, and support. Moreover, Cocca and Cocca (2019) found out that integrative motivation is positively correlated with language learning achievement by inspiring learners to use language efficiently, and that it is also related to learners’ culture. Liu (2021) stated that second language learning is significantly affected by integrative rather than instrumental correlation. He argued that motivated learners attempt to find chances to develop their language skills.

Alternatively, Gardner and Lambert (1972, p. 150) stated that “instrumental orientation is considered a desire to gain social recognition or economic advantages through knowledge of a foreign language.” Cheng et al. (2014) claimed that instrumental motivation can negatively affect test results. Sallang and Ling (2019) found a relationship between providing constructive feedback to students and instrumental motivation in language learning as it develops learners’ motivation in performing tasks. Safotso and Tompte’s (2018) study revealed that learners with high levels of instrumental motivation are more interested in communicative approach in language learning.

Generally, Gardner (2001) mentioned that positive perspectives toward the educational environment and instrumental and integrative-oriented motivations significantly influence students’ thoughtfulness. Khalid (2016) also stated that instrumental and integrative motivations in language learning are correlated with socioeconomic situations. However, he claimed that perspectives on language learning can be influenced by integrative and instrumental orientations. Sediqifar and Khaleghizadeh’s (2016) study revealed that learners’ integrative motivation is positively correlated with their academic achievement. Nevertheless, their study did not improve the significant correlation between learners’ instrumental motivation and language learning achievements. One of the causes of EFL learners’ motivation appears to be technology use. In recent times, numerous investigations have been conducted on this issue (Deeler and Gray, 2000).

### The Role of Educational Technology in English as a Foreign Language Learners’ Motivation

Numerous motivation investigations have been done in conventional instructional environments, i.e., face-to-face educational contexts. There are some limitations in traditional educational contexts that probably affect learners’ motivation, such as doing tasks with insufficient resources introduced in educational environments (Wolters, 2011). Despite the importance of educational technology in fostering learners’ motivation, a few investigations have thoroughly examined the significance of motivation in the digital learning context. In a study, found that learners are generally inclined to have higher levels of motivation when they are using digital instruments for foreign language learning. He also argued that learners in technology-based contexts show their integration with the community as they can prevail over loneliness and can be familiarised with various societies and cultures. Yang and Wu (2012) asserted that educational technology is required to have well-defined purposes that provoke learners’ attention and, therefore, enhance engagement and motivation. Stepp-Greany (2002) revealed that using multimedia programmes can enhance learners’ enjoyment and motivation. Ushida (2005) also stated that optimism and motivation in foreign language learning significantly correlate with learners’ academic success in online language learning. Genc Ilter (2009), in her study, revealed various digital tools, including videos, the internet, computers, projectors, and multi-media differently affect learner motivation in foreign language contexts. She argued that learners’ levels of motivation are significantly correlated with their employ of technology. Golonka et al. (2014), in another study, pointed out that using varieties of digital instruments in education can contribute to the improvement of learners’ interest and motivation. They also mentioned that technology increases input flooding opportunities, helps learners engage in academic contexts, and assists learners in interacting with native speakers. Çelik and Aytin (2014) declared that learners’ positive attitudes toward learning and their intrinsic motivation are influenced by the employment of authentic materials and interactive language learning contexts using technology. Moreover, the research provided by Kalanzadeh et al. (2014) showed that teacher and learner rapport is positively and significantly affected by technology, and that this enhances learner motivation and teacher work engagement. Moreover, web-based supplementary instruments are critical in improving learners’ motivation to communicate with peers in blended educational contexts (Hirata, 2018). Sauro and Sundmark (2016) also asserted that MALL and social media have facilitated EFL learners’ academic achievements and increased their engagement and intrinsic motivation in foreign language learning. In a study, Honarzad and Rassaei (2019) revealed that learners’ self-sufficiency, self-efficacy, and motivation are significantly affected by technology-based language learning tasks. Furthermore, Gürkan (2018) examined the practicality of vocabulary learning applications and inferred that the features, such as attractiveness, effectiveness, and usefulness of video, and graphics are significant in applications to increase learner motivation. Azmi (2017) also suggested that integrating instructional technology in the EFL context increases independent education, amplifies learning achievements, and motivates learners. He mentioned that podcasts and digital videos in classroom contexts are valuable to increase learner motivation and engagement. He argued that inefficient use of educational technology is expected to be a challenging issue for technology to increase learner autonomy and motivation. Kawinkoonlasate (2020) demonstrated that educational technology augments social interactions among learners, and enhances learner motivation to the instructed material. Alghasab (2020) has shown that educational technology broadens learner views about language learning, and can transform the learning context into a more favourable one to raise learner motivation and engagement. Tavakoli et al. (2019) found the positive impact of CALL-mediated task-based learning on students’ engagement and motivation in foreign language reading. They also argued that CALL is not the sole variable that increases learners’ motivation. They mentioned that the availability, clarity, and attractiveness of the materials are the other reasons for the increases in learner motivation. Song and Bonk (2016) investigated self-regulation in the online learning context. They found out that an online learning context can intensify learners’ motivation in self-regulated language learning. Berenji and Saeidi (2017) also revealed that technology-mediated instruction, through using Telegram, leads to cognitive scaffolding and the enhancement of motivation and academic achievement. To justify their results, they used an activity learning theory which “focuses on learning as an interactive activity and interaction of human activity and consciousness within an environmental context relevant to it.” Aysu (2020), in a study, found that learners have more integrative than instrumental motivation in technology-based contexts. He also maintained that motivated learners in online contexts have some features, such as “being goal-directed, expending effort, being persistent, being attentive, having desires (wants), exhibiting positive effect, being aroused, having expectancies, demonstrating self-confidence (self-efficacy), and having reasons (motives)” (p. 2). De Souza et al. (2021) also found a significant and positive relationship between teacher belief in the integration of digital tools in educational contexts and the engagement and motivation of learners in language learning. They also found that learners’ motivation in technology-based language learning is not correlated with their beliefs in the important position of teachers in their life.

Few studies have been done on the effect of technology-mediated instruction on EFL learners’ motivation in Chinese contexts. Peng and Fu (2021) showed that blended instruction can positively affect the association between learners’ intrinsic and extrinsic motivation and academic performance. They also found out that technology-based instruction expedites learners’ proficiency and positive emotions. However, their findings suggested that Chinese EFL learners’ extrinsic motivation is more substantial than intrinsic motivation. Therefore, they suggested the development of intrinsic motivation through using practicable methods, such as the employment of competition-based learning, game-based learning, and digital storytelling approach, to improve learners’ intrinsic motivation. Liu et al. (2019) investigated the motivation types in using smartphone dictionaries. They classified foreign language learning motivations into customisation, learning, and utility categories. They found that most lower-proficient learners tend to use e-dictionary for customisation; that is, they use dictionaries based on their needs. On the other hand, higher-proficient learners use dictionaries for learning a language, and their motivation does not include learning for exams or meeting their needs. Yang (2020), in another study, found out that the learners’ motivation is affected by task difficulty, mobile technologies, the attractiveness of materials, and motivational objectives of the mobile applications. However, they did not find any significant difference between WeChat as a social media and the English language learning motivation. He also argued that those who have negative motivation toward MALL could not take advantage of it, which confirms the theory that differentiated feelings toward MALL indirectly impact language learning motivation. In another study on Chinese learners, Li (2021) found that game-based vocabulary learning application significantly affects learners’ self-confidence, intrinsic motivation, and vocabulary achievement. He argued that game-based learning application enhances enjoyment which then triggers the enhancement of learners’ learning motivation. Teng and Wang (2021) compared learning management systems (LMS) and social networking systems and their effect on learners’ emotional engagement. Using Superstar-Xuexitong and WeChat, they found out that LMS significantly arouses learner motivation.

## Implications

This review investigated the related literature on the effect of educational technology on learners’ motivation for foreign language learning. This review implicates that learners should be aware of their motivation in online language learning by controlling, adjusting, and regulating their feelings in online language learning contexts. Autonomous Learners should develop their motivation to use digital tools to promote their academic performance. They can choose their own digital tools based on their language proficiency level and enjoy their motivational objectives in language learning. This review suggests that learners use technology as the source of learning material for their language learning. Moreover, inspecting learner requirements should be one of the fundamentals of instruction in an online language educational environment to attain instructional results. To do that, they are required to reconstruct and modify the materials offered to them. L2 instructors should find authentic and interactive materials to develop learners’ positive attitudes and their motivation for increasing positive emotions. This can diminish learners’ cognitive load in online learning and increase their concentration on language learning contexts. They may also change their methodology by taking motivation into account in their instruction. They can offer warm-up activities for educational contexts and brainstorm learners to increase motivation in online contexts. The projects, lectures, conferences, and workshops may demotivate learners and put extra stress on learners in online environments. To motivate learners, familiarising learners with questions of the tests can be helpful, and teachers can change their course assessment in online and traditional classrooms. In addition, they may evaluate learners’ achievements and increase operational language learning by considering the factors which trigger learners’ motivation. Teachers can manage the time of classrooms regularly along with learner engagement to arouse motivation. Instructors are required to decrease learners’ foreign language anxiety and disengagement. In addition, they need to increase learners’ motivation irrespective of educational problems in language learning environments to enrich L2 learning experiences. They should talk to learners about their internal and external motivations in online contexts to be aware of learners’ personality traits, helping them to engage enthusiastically in online learning contexts. They can make their class interactive and motivated by asking challenging questions throughout the class. Through asking and answering questions, learners can be more engaged during the course and learn information efficiently. Providing a competitive educational context through quizzes augments learner engagement and motivation. Unplanned quizzes are predominantly important for inspiring less engaged and unready learners. Collaboration is another way for teachers to increase learner motivation. Learners tend to engage in classrooms when they cooperate with each other on class projects. Teachers can provide learners with some video files like TED videos in online classrooms, and they can discuss the effect of motivation on inspiring individuals’ performance. Teachers can provide models to learners by playing videos about thoughtful individuals who are motivated and successful in their life despite their disabilities.

Internet age allows information users to study and communicate on the internet at any time. Along with it, Massive Open Online Courses (MOOCs), a large scale open network courses, and diversity of new teaching methods develops vigorously (Rolfe, 2015). There is no doubt that the beginning of the MOOC development brings superiority and greatly promotes the reform and development of course teaching in colleges and universities in our country, but it also inevitably expose some problems. The Small Private Online Courses (SPOCs), a small scale restricted online course, also starts to attract people’s attention, and the opening of the SPOC mode makes profound changes in the course teaching. Therefore, teachers can use MOOC and SPOC to motivate learners for learning.

Teacher educators and mentors can provide some techniques for teachers to select network-based materials based on learners’ needs analysis and motivations. They can hold workshops for in-service teachers to encourage them to use technology in education. It is also suggested that teacher educators highlight interaction tools, like mobile applications, which motivate learners and promote interaction among learners. This review recommends that teacher educators should have a positive view toward teachers and learners, and that they should provide well-organised and inspiring teaching methodologies which can construct a motivation for language learning and engagement in the classroom. Teacher educators should provide elbow support to enhance paedagogical skills in online instruction. They should develop confidence and competence among in-service teachers to entice learners’ interests and engage them in the learning process.

This review can also stimulate educational policymakers to consider EFL learners’ motivation in online educational contexts. They should provide computer labs, projectors, CD and DVD players for instructors and help them use digital tools. They can hold academic workshops to help teachers increase motivation among learners. They can provide internet-based facilities and positive learning contexts for increasing positive behaviours and motivations among learners. Therefore, this review implicates that policymakers can change their opinion about using technology as the authentic material in English classrooms. The importance of motivation may make consultants expand their agendas to diagnose learners’ demotivating reasons and the obstacles they cope within language learning.

Future studies need to be done on learners’ motivation in online contexts in numerous cultural backgrounds. Some investigations need to be done on the effect of educational technology on learners’ mindsets and other positive emotions. Online learning for EFL learners might not be appropriate to all learners. Therefore, future research should be devoted to the impact of gender, background, age, and earlier online learning experience on learner motivation. Furthermore, the proficiency level of EFLs and its effect on motivation to learn in online learning should be considered for the future. Furthermore, case and phenomenological investigations which provide us the reasons behind learner motivation in online learning contexts are required to be done. The effect of different digital tools on learners’ motivations and positive and negative emotions need to be studied. It is also desirable to investigate the impact of technology on improving other language characteristics, such as attitude and emotional intelligence.

## Author Contributions

The author of the current study independently designed and drafted the manuscript.

## Conflict of Interest

The author declares that the research was conducted in the absence of any commercial or financial relationships that could be construed as a potential conflict of interest.

## Publisher’s Note

All claims expressed in this article are solely those of the authors and do not necessarily represent those of their affiliated organizations, or those of the publisher, the editors and the reviewers. Any product that may be evaluated in this article, or claim that may be made by its manufacturer, is not guaranteed or endorsed by the publisher.
